# Adult acute lymphoblastic leukaemia: a study of prognostic features and response to treatment over a ten year period.

**DOI:** 10.1038/bjc.1986.32

**Published:** 1986-02

**Authors:** R. E. Marcus, D. Catovsky, S. A. Johnson, W. M. Gregory, J. G. Talavera, J. M. Goldman, D. A. Galton

## Abstract

Between 1974 and 1984 69 adults with acute lymphoblastic leukaemia (ALL) were treated with two different protocols. Fifty-four (78%) of the patients entered complete remission (CR); 27 of these then received a consolidation protocol consisting of daunorubicin, cytosine arabinoside and 6-thioguanine, followed by two courses of intravenous methotrexate 500 mg m-2 with folinic acid rescue. All patients received intrathecal methotrexate and cranial irradiation (24 Gy) followed by maintenance therapy with 6-mercaptopurine and methotrexate for at least 2 years. The median survival for all patients was 23 months from the time of presentation with an actuarial 5-year survival of 21%. The actuarial chance of surviving 5 years in CR for patients receiving the consolidation protocol was 38% compared to 19% for patients receiving no consolidation (P = NS). Only patient age and white cell count at presentation were found to influence the chance of achieving CR and the chance of overall survival. The presence or absence of c-ALL antigen did not influence prognosis. Patients younger than 35 years with low white cell counts at presentation (less than 10 X 10(9)1(-1] had a particularly good prognosis but no patient with T-ALL and no patient older than 50 years old at diagnosis survived more than 18 months.


*Br. J. Cancer* (1986), **53**, 175–180

### Adult acute lymphoblastic leukaemia: A study of prognostic features and response to treatment over a ten year period

R.E. Marcus, D. Catovsky, S.A. Johnson, W.M. Gregory, J.G. Talavera, J.M. Goldman & D.A.G. Galton

*MRC Leukaemia Unit, Royal Postgraduate Medical School, Ducane Road, London W12 0HS, UK.*

**Summary** Between 1974 and 1984 69 adults with acute lymphoblastic leukaemia (ALL) were treated with two different protocols. Fifty-four (78%) of the patients entered complete remission (CR); 27 of these then received a consolidation protocol consisting of daunorubicin, cytosine arabinoside and 6-thioguanine, followed by two courses of intravenous methotrexate $500 \mathrm{mg\,m^{-2}}$ with folinic acid rescue. All patients received intrathecal methotrexate and cranial irradiation (24 Gy) followed by maintenance therapy with 6-mercaptopurine and methotrexate for at least 2 years. The median survival for all patients was 23 months from the time of presentation with an actuarial 5-year survival of 21%. The actuarial chance of surviving 5 years in CR for patients receiving the consolidation protocol was 38% compared to 19% for patients receiving no consolidation ($P$=NS). Only patient age and white cell count at presentation were found to influence the chance of achieving CR and the chance of overall survival. The presence or absence of c-ALL antigen did not influence prognosis. Patients younger than 35 years with low white cell counts at presentation ($<10\times10^9\,\mathrm{l}^{-1}$) had a particularly good prognosis but no patient with T-ALL and no patient older than 50 years old at diagnosis survived more than 18 months.

In children with acute lymphoblastic leukaemia (ALL) survival is adversely affected by a number of features at presentation including high white cell count (WBC), age over ten years, male sex, and the presence of a mediastinal mass (Simone *et al.*, 1975; Chessells *et al.*, 1981). Anaemia, thrombocytopenia, the absence of the common c-ALL antigen (CALLA) and the morphological FAB subtype L2 have also been shown to be associated with poor prognosis (Greaves *et al.*, 1981; Sallan *et al.*, 1980). The overall five year survival in children is ~50% (Chessells *et al.*, 1977; Hagbin *et al.*, 1980) although some centres using more intensive protocols report better results (Henze *et al.*, 1982; Lampert *et al.*, 1984).

The outlook for adults with ALL is worse than that of younger patients. In adults the 5-year survival is <30% in most series (Lister *et al.*, 1978; Jacobs & Gale, 1984; Henderson *et al.*, 1979). Furthermore it is not clear whether the adverse prognostic features in childhood leukaemia also apply to adults, or whether intensive consolidation therapy given in remission will improve prognosis. In this paper we report the results of treating adults with ALL with two sequential protocols used between 1974 and 1984. We correlated their response to treatment with specific features at presentation.

Correspondence: D. Catovsky.
Received 19 August 1985; and in revised form, 25 October 1985.

### Materials and methods

### *Patients*

Sixty-nine patients, 46 men and 23 women, aged from 16–66 years (mean 30.2 years) were treated over the 10-year period 1974 to 1984. The diagnosis was based on the examination of the blast cells in Romanowsky stained films and cells were classified according to the criteria of the FAB group (Bennett *et al.*, 1981). Cytochemical stains for Sudan black, myeloperoxidase, PAS and acid phosphatase were also carried out. Terminal deoxynucleotidyl transferase was measured initially by a biochemical assay and later by immunofluorescence (Bollum, 1979), Cell surface marker studies were performed as follows: T-cell ALL was diagnosed initially on the basis of E-rosetting with sheep red cells and more recently by specific anti-T cell monoclonal antibodies. The presence of the CALLA was assessed initially by heterologous antiserum (Greaves *et al.*, 1975) and later by the J5 monoclonal antibody (Ritz *et al.*, 1980). Specific anti-human immunoglobulin sera were used to make the diagnosis of B-cell ALL.

The pre-treatment clinical and haematological characteristics for all patients are summarised in Table I. The incidence of the FAB L2 subgroup increased with age (Figure 1); L2 morphology was found in 27 of the 49 patients below the age of 35 (58%) and in 13 out of 18 patients over 35 (77%) ($P<0.05$). There was no significant relationship between the proportion of patients with common-ALL antigen (CALLA) positive cells and age.

© The Macmillan Press Ltd., 1986

**Table I** Patient characteristics and response to treatment.

| | *No.* | *CR(%)* | *Median survival (months)* Remitters | *Median survival (months)* All |
|---|---|---|---|---|
| ALL | 69 | 54 (78) | 21 | 23 |
| Sex | | | | |
| M | 46 | 35 (76) | 22 | 22 |
| F | 23 | 19 (83) | 23 | 24 |
| *WBC count at presentation* ($\times 10^9 l^{-1}$) | | | | |
| 0–10 | 31 | 28 (90) | 26 | 29 |
| 11–100 | 23 | 16 (69)[a] | 22 | 18[a] |
| 100+ | 15 | 10 (66) | 11[b] | 12[b] |
| *AGE (years)* | | | | |
| >50 | 11 | 2 (18) | 6 | 6 |
| <50 | 58 | 54 (90)[b] | 24[b] | 26[b] |
| *FAB class* | | | | |
| L1 | 26 | 24 (92) | 21 | 25 |
| L2 | 40 | 27 (68)[a] | 22 | 20 |
| L3 | 1 | 1 (100) | 3 | 4 |
| *Phenotype* | | | | |
| CALLA | | | | |
| +VE | 36 (66) | 28 (78) | 21 | 25 |
| −VE | 13 (19) | 8 (61) | 23 | 18 |
| T-ALL | 5 (9) | 4 (80) | 9[a] | 8 |
| *Therapy trial* | | | | |
| Standard | | | | |
| ALL | 36 | 27 (74) | 20 | 19 |
| ALL+DAT | 33 | 27 (78) | 21 | 26 |

[a]$P<0.05$, [b]$P<0.01$.

**Figure 1** Distribution of the two major FAB types of ALL in relation to the patients' age at presentation (■) L1; (□) L2.

There was no correlation between the phenotype of the blast cells (CALLA+ or CALLA−) and morphology (FAB L1 or L2).

### Treatment protocols

*A. Protocol in use from 1974 to 1978*

*Induction* Vincristine (1.4 mg m$^{-2}$, max. 2 mg) and prednisolone (40 mg m$^{-2}$).

*Consolidation* L-asparaginase (10 000 u m$^{-2}$) or cyclophosphamide (600 mg m$^{-2}$ × 1) with vincristine (1.4 mg m$^{-2}$ × 1) cytosine arabinoside (100 mg m$^{-2}$) daily and prednisolone (40 mg m$^{-2}$) daily for 5 days (COAP).

*Maintenance* Continuous methotrexate (15 mg mg$^{-2}$ daily for 3 to 5 days and 6-mercaptopurine (70 mg m$^{-2}$) for 2 years with precise dosage adjusted in accordance with the blood counts.

*B. Protocol in use from 1978 to 1982*

*Induction* Vincristine and prednisolone with L-asparaginase as above.

*Consolidation* Daunorubicin 50 mg m$^{-2}$ daily for 3 days, cytosine arabinoside 100 mg m$^{-2}$ for 7 days, 6-thioguanine 150 mg m$^{-2}$ for 7 days (DAT) followed by methotrexate 500 mg m$^{-2}$ with folinic acid rescue repeated on a second occasion after peripheral blood recovery.

*Maintenance* Continuous methotrexate and 6-mercaptopurine as above for 2 years with re-induction every 12 weeks with vincristine (1.4 mg/weekly × 3) and prednisolone 40 mg m$^{-2}$ for 3 weeks.

*C. Protocol in use from 1982 to 1984*

*Induction* Vincristine, prednisolone and L-asparaginase with adriamycin 50 mg m$^{-2}$ weekly for 3 weeks and cyclophosphamide 600 mg m$^{-2}$ weekly for 3 weeks.

*Consolidation* DAT followed by high dose methotrexate × 2 as above.

*Maintenance* As per 1978–1982 schedule above plus adriamycin 50 mg m$^{-2}$ × 1 and cyclophosphamide 750 mg m$^{-2}$ × 1 as part of 12 weeks re-induction cycle.

All patients received central nervous system prophylaxis with fractionated cranial irradiation (24 Gy) and intrathecal methotrexate (10 mg m$^{-2}$, 12 mg maximum) on eight occasions.

Patients are divided into two treatment groups for the purpose of this analysis: (A) those who received standard ALL therapy (1974–1978) ($n=27$) and (B) those who received DAT consolidation therapy and high dose intravenous methotrexate (1978–1984) ($n=27$).

*Statistical methods*

Survival and remission curves were calculated by means of standard life table techniques (Kaplan & Meier, 1958). Statistical significance was determined by the log rank method (Peto *et al.*, 1977). The significance of prognostic factors in determining the duration of overall survival and of CR was evaluated by stepwise logistic regression methods using Cox's proportional hazards model (Cox, 1972).

### Results

*Remission induction rates*

Fifty-four (78%) of the patients entered CR, including four of the five patients with T-ALL. The CR rate decreased with age: only two of the 11 patients over the age of 50 years and only nine of the 18 patients older than 35 years achieved CR ($P<0.01$). The 15 patients who did not achieve CR all died within 6 months of presentation either with resistant disease or from infection. There was no difference in CR rates between the two treatment groups or between men and women. Twenty-eight (90%) out of 31 patients with presenting WBC counts less than $10\times10^9 l^{-1}$ entered CR whereas only 26 out of 38 (69%) patients with higher presentation WBC counts entered CR ($P<0.05$). Twenty-four out of 26 patients (92%) with L1 morphology entered CR compared to 28 out of 42 (68%) with L2 morphology ($P<0.05$). No differences were recorded in CR rate between CALLA +ve and CALLA −ve patients.

*Survival of patients in CR*

The median survival for all patients entering CR was 23 months. The median follow-up time is 76 months with 32% of patients remaining in continuous CR (Figure 2). No patient who remained in first CR for more than 5 years has subsequently relapsed. Of the 34 patients who relapsed 31 died within 1 year of relapse regardless of whether or not a second CR was achieved. There are three long-term survivors in second or third remission more than 1 year after first relapse. The four patients with T-ALL who entered CR all relapsed and died within 1 year of entering CR. The one patient with L3 ALL entered CR but relapsed with CNS disease within 3 months and died with CNS and bone marrow relapse. For patients in CR there were no significant differences

**Figure 2** Actuarial disease-free survival for the 54 patients who achieved complete remission. The plateau is at 32%. Diagonal marks represent individual patients who survive in CR.

in duration of CR between patients with L1 or L2 morphology, CALLA+ or CALLA− phenotype, aged less or greater than 35 years or between men and women.

Patients with the lowest presenting white cell count had the best outlook after achieving CR. Patients with presenting WBC counts $<10\times10^9 l^{-1}$ had a median duration of CR of 26 months compared to 22 months for patients presenting with WBC count between 10 and $100\times10^9 l^{-1}$ and 11 months for patients presenting with WBC counts $>100\times 10.9 l^{-1}$ ($P<0.01$) (Figure 3).

**Figure 3** Actuarial disease-free survival for the 54 patients who achieved CR analysed by WBC count at presentation. Diagonal marks represent individual patients who survive in CR. WBC ($\times10^9 l^{-1}$) 0–10(-----) $n=28$; 11–100(——) $n=16$; 100+(——) $n=10$. $P<0.05$.

*Factors influencing overall survival*

The median survival for all patients from the date of presentation was 23 months with median follow-up of 77 months. The factors at presentation that influence survival can be divided into two categories: those that affect the chances of attaining CR, and hence influence survival but have no relevance once CR has been achieved, and those that are relevant both to the achievement of and survival after CR.

Two factors of the first type were found, namely age and blast cell morphology. Patients under the age of 35 survived longer than older patients ($P<0.01$, Figure 4), as did patients with L1 morphology when compared to L2 morphology ($P<0.05$). However, the significance of the influence of morphology was not confirmed by multivariate analysis, which found age to be the more dominant factor, and explains the survival advantage for the L1 patients as due to their younger mean age. There were no long-term survivors among patients presenting over the age of 50 years.

**Figure 4** Actuarial survival for all 69 patients analysed by age at presentation. Diagonal marks represent surviving patients. (——) 15–35 y, $n=51$; (-----) 35–66 y, $n=18$. $P<0.01$.

Only WBC at presentation influenced survival by affecting both the chance of achieving CR and the survival after CR was attained.

Forty-one percent of patients with the lowest WBC count ($<10\times10.9\,l^{-1}$) remained in continuous CR at the time of writing compared to under 20% for all patients presenting with WBC count $>10\times10.9\,l^{-1}$ ($P<0.05$). It is possible to identify a subgroup of patients under the age of 35 years with presenting WBC counts of $<10\times10^9\,l^{-1}$ who had an overall median survival from the time of diagnosis of 32 months and an actuarial five year survival of 42% compared to 23 months and 21% respectively for all other patients ($P<0.05$). The independent effects of age and white cell count on survival was established by multivariate regression analysis. The surface phenotype (CALLA+ve or CALLA−ve) was not found to influence the duration of CR in this series.

*Effect of consolidation chemotherapy*

Twenty-seven patients received standard consolidation therapy and 27 intensive therapy in remission. There was no difference in the pretreatment characteristics of the two groups, and no difference in the overall CR rates (Table II). The median disease-free survival was 20 months for patients receiving standard consolidation therapy and 21 months for patients receiving intensive therapy. The predicted actuarial chances of remaining in CR (Figure 5) at 5 years were 18% (standard chemotherapy) and 38% (intensive consolidation) with median follow-up times of 93 and 34 months respectively ($P=$NS).

**Table II** Characteristics of patients receiving two treatment protocols.

| | *Patients receiving DAT consolidation* | *Patients receiving standard ALL therapy* |
|---|---|---|
| Sex | | |
| M | 19 (57%) | 27 (75%) |
| F | 14 (43%) | 9 (25%) |
| Mean WBC | | |
| ($\times10^9\,l^{-1}$) | 43 (0–96) | 69 (1–160) |
| Mean age (y) | 31 (15–66) | 30 (16–64) |
| FAB subtype | | |
| L1 | 11 (36%) | 15 (58%) |
| L2 | 19 (64%) | 21 (42%) |
| CALLA | | |
| +VE | 22 (81%) | 14 (64%) |
| −VE | 5 (19%) | 8 (36%) |
| Total | 27 (50%) | 27 (50%) |

There was no statistically significant difference between any of the patient pretreatment characteristics for the two treatment protocols.

**Figure 5** Actuarial disease-free survival for 54 patients who achieved CR analysed by type of consolidation and/or maintenance chemotherapy in remission. Diagonal marks represent patients surviving in CR. (——) DAT consolidation, $n=27$; (-----) standard ALL therapy, $n=27$. $P=$n.s.

### Discussion

Most protocols used to treat adults with ALL are based on the regimens employed with success in childhood, but despite the achievement of CR in the majority of cases most adults with ALL relapse and die within 4 years. This contrasts with the long disease-free survival seen in children (Jacobs & Gale, 1984). The CR rate in the patients reported here is similar to those reported in several recent studies (Baccarani *et al.*, 1982; Hoelzer *et al.*, 1984; Schauer *et al.*, 1983; Aviles *et al.*, 1983; Gottlieb *et al.*, 1984). Lower CR rates were observed in older patients and those with high presenting WBC counts.

Early intensive consolidation has been reported to improve survival in children (Henze *et al.*, 1982; Lampert *et al.*, 1984) and our own programme of DAT consolidation given early in remission was designed to achieve similar benefit in adults. In the event a minor improvement was observed in the actuarial survival with the more intensive regimen, but this was not significant. Longer-term follow-up and larger numbers of patients are needed to enable firm conclusions to be drawn.

Univariate analysis of the individual patient characteristics suggested that L2 morphology, increasing age and high WBC influence the chances of entering CR and of survival. The multivariate analysis showed, however, that the adverse effect of L2 morphology could not be separated from the effect of age. There are conflicting reports on the prognostic significance of the morphological subtype (Leiment *et al.*, 1980; Brearley *et al.*, 1975) and the influence of age may be more important. This analysis further showed that age at diagnosis and white cell count are the only two variables to influence survival at the time of presentation. For patients who achieved CR only the WBC at presentation had an effect on outcome; we found that the log 10 of the leucocyte count at presentation had the greatest influence on survival. This suggests that it is the tumour burden at the time of diagnosis which affects the chance of long-term survival. These findings are similar to other recently reported series (Mertelsman *et al.*, 1982). Hoelzer and co-workers have shown that disease-free survival in their patients with T-ALL was comparable to that seen in patients with common ALL (Hoelzer *et al.*, 1984) but this was not our experience with smaller numbers of patients.

We identified a subgroup of patients under the age of 35 with presenting WBC less than $10 \times 10^9 l^{-1}$ who have a particularly favourable outlook with conventional chemotherapy; this may improve further with more intensive treatment regimens. It might not therefore be appropriate to offer to this group of patients allogeneic bone marrow transplant (BMT) in first remission in view of the relatively high mortality of BMT and the relatively high relapse rates for those who survive the procedure (Barrett *et al.*, 1985). More intensive induction therapy may benefit older patients in whom CR rates were low, since their survival once in remission was not affected by age. Those patients presenting with a higher WBC than $10 \times 10^9 l^{-1}$ had a poor prognosis, whether or not CR is achieved. Improved survival might be obtained by intensification both of induction and consolidation therapy. The prognosis in those young patients whom we have defined as having a particularly poor outlook could perhaps be improved by allografting or by autografting in first remission.

We conclude that the accurate identification of the different prognostic groups in adult ALL, which seem to differ from those in children, should facilitate selection of the appropriate treatment and improve the long-term outlook.

REM and SAJ were supported by the Leukaemia Research Fund. WMG is currently working at the Imperial Cancer Research Fund.

**References**

AVILES, A., SINCO, A., RIVERA, R., AMBRIZ, R., HERRERA, J.G. & PIZZUTO, J. (1983). Treatment of adult lymphoblastic leukemia with adriamycin vincristine and prednisolone. *Med. Ped. Oncol.*, **11**, 141.

BACCARANI, M., CORBELLI, G., AMADORI, A. *et al.* (1982). Adolescent and adult acute lymphoblastic leukemia – prognostic features and outcome of therapy – a study of 293 patients. *Blood*, **60**, 677.

BARRETT, A.J., JOSHI, R. & TEW, C. (1985). How should acute lymphoblastic leukaemia relapsing after bone-marrow transplantation be treated? *Lancet*, **i**, 1188.

BENNETT, J.M., CATOVSKY, D., DANIEL, M.T. & 4 others. (1981). The morphological classification of acute lymphoblastic leukaemia – concordance between observers and clinical correlations. *Br. J. Haematol.*, **47**, 553.

BOLLUM, F.S. (1979). Terminal deoxynucleotidyl transferase as a hemopoietic cell marker. *Blood*, **54**, 1203.

BREARLEY, R.C., JOHNSON, S.A. & LISTER, T.A. (1975). Acute lymphoblastic leukaemia in adults: clinico-pathological correlation with the French–American–British classification. *Eur. J. Cancer*, **15**, 904.

CHESSELLS, J., HARDISTY, R.M., RAPSON, R.M. & GREAVES, M.F. (1977). Acute lymphoblastic leukaemia in children, classification and prognosis. *Lancet*, **i**, 1307.

CHESSELLS, J.M. (1982). Acute lymphoblastic leukaemia. *Semin. Hematol.*, **19**, 155.

COX, D.R. (1972). Regression models and life tables. *J. Roy. Stat. Soc.*, **34**, 187.

GOTTLIEB, A.J., WEINBERG, V., ELLISON, R.R. *et al.* (1984). Efficacy of daunorubicin in the therapy of acute lymphocytic leukemia in adults – a prospective randomised trial by Cancer and Leukemia group B. *Blood,* **64,** 267.

GREAVES, M.F., JANOSSY, G., PETO, J. & KAY, H.E.M. (1981). Immunologically defined subclasses of acute leukemia in children: their relationship to presentation features and prognosis. *Br. J. Haematol.,* **48,** 179.

GREAVES, M.F., BROWN, G., RAPSON, N. & LISTER, T.A. (1975). Antisera to acute lymphoblastic leukaemia cells. *Clin. Immunol. Immunopathol.,* **4,** 67.

HAGBIN, M., MURPHY, M.L., TAN, C.C. *et al.* (1980). A long term follow up of children with acute lymphoblastic leukemia treated with an intensive chemotherapy regimen. *Cancer,* **46,** 241.

HARDISTY, R.M., TILL, M.M. & PETO, J. (1981). Acute lymphoblastic leukaemia: four year survivals old and new. *J. Clin. Path.,* **34,** 249.

HENDERSON, E.S., SCHARLAU, C., COOPER, M.R. *et al.* (1979). Combination chemotherapy and radiotherapy for acute lymphoblastic leukemia in adults: results of the CALGB protocol 7113. *Leukemia Res.,* **3,** 395.

HENZE, G., LANGERMANN, H. J., FENGLER, R. *et al.* (1982). Therapiestudie BFM769/81 zur Behandlung der akuten lymphoblatischen Leukaemia bei Kindern und Jugendlichen: Intensivierte Reinduktionstherapie fuer Patientgruppen mit interschiedlichen Rezidivrisiko. *Klin. Padiat.,* **194,** 195.

HOELZER, D., THIEL, E., LOEFFLER, H. *et al.* (1984). Intensified therapy in acute lymphoblastic leukemia and acute undifferentiated leukaemia in adults. *Blood,* **64,** 38.

JACOBS, A.D. & GALE, R.P. (1984). Recent advances in the biology and treatment of acute lymphoblastic leukemia in adults. *New Engl. J. Med.,* **311,** 1219.

KAPLAN, E.S. & MEIER, P. (1958). Non-parametric estimation from incomplete observations. *American Stat. Assoc. J.,* **53,** 457.

LAMPERT, F., HENZE, G., LANGERMANN, H.J., SCHELLONG, G., GADNER, H. & RIEHM, H.J. (1984). Acute lymphoblastic leukemia: current status of therapy in children. In *Recent Results Cr. Res.,* **93,** Thiel, E. & Thierfelder, S. (eds) p. 159. Springer-Verlag: Berlin and Heidelberg.

LEIMENT, J.T., BURNS, C.P., WITTSE, C.G., ARMITAGE, J.O. & CLARKE, W.R. (1980). Prognostic influences of pretreatment characteristics in adult acute lymphoblastic leukemia. *Blood,* **56,** 510.

LISTER, T.A., WHITEHOUSE, J.M., BEARD, M.E.J. *et al.* (1978). Combination chemotherapy in 130 patients with acute lymphoblastic leukaemia in adults. *Br. Med. J.,* **1,** 199.

MERTELSMANN, R., MOORE, M.A.S. & CLARKSON, B.D. (1982). Leukemia cell phenotype and prognosis. An analysis of 519 adults with acute leukemia. *Blood Cells,* **8,** 561.

PETO, R., PIKE, M.C., ARMITAGE, P. *et al.* (1977). Design and analysis of randomised clinical trials requiring prolonged observation of each patient. *Br. J. Cancer,* **35,** 1.

RITZ, J.M., PESANDO, J.M., NOTIS-McCONARTY, J., LAZARUS, H. & SCHLOSSMAN, S.F. (1980). A monoclonal antibody to human lymphoblastic leukaemia antigen. *Nature,* **283,** 583.

SALLAN, S.E., RITZ, J., PESANDO, J. *et al.* (1980). Cell surface antigens and prognostic implications in children with acute lymphoblastic leukemia. *Blood,* **55,** 395.

SCHAUER, P., ARLIN, Z.A., MERTELSMANN, R. *et al.* (1983). Treatment of acute lymphoblastic leukemia in adults; results of the L10 and L10M protocols. *J. Clin. Oncol.,* **1,** 462.

SIMONE, J.V., VERZOSA, M.S. & RUDY, T.A. (1975). Initial features and prognosis in 363 children with acute lymphoblastic leukemia. *Cancer,* **36,** 2099.


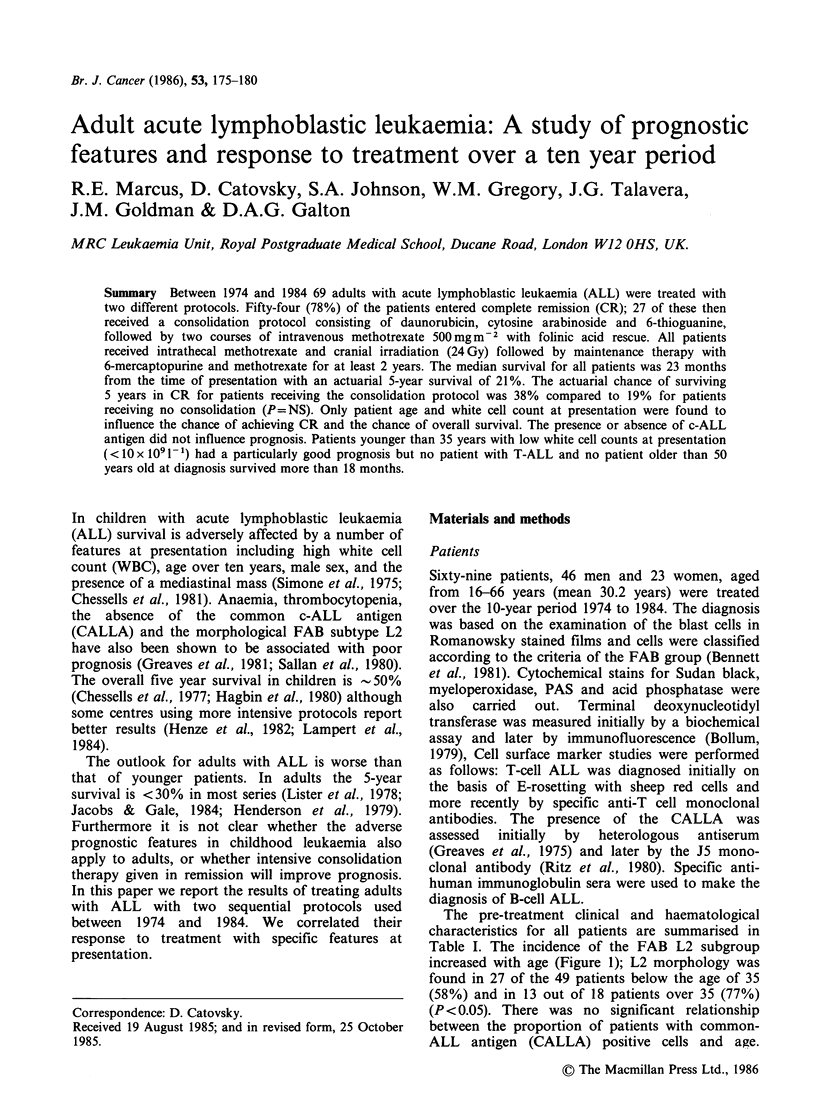

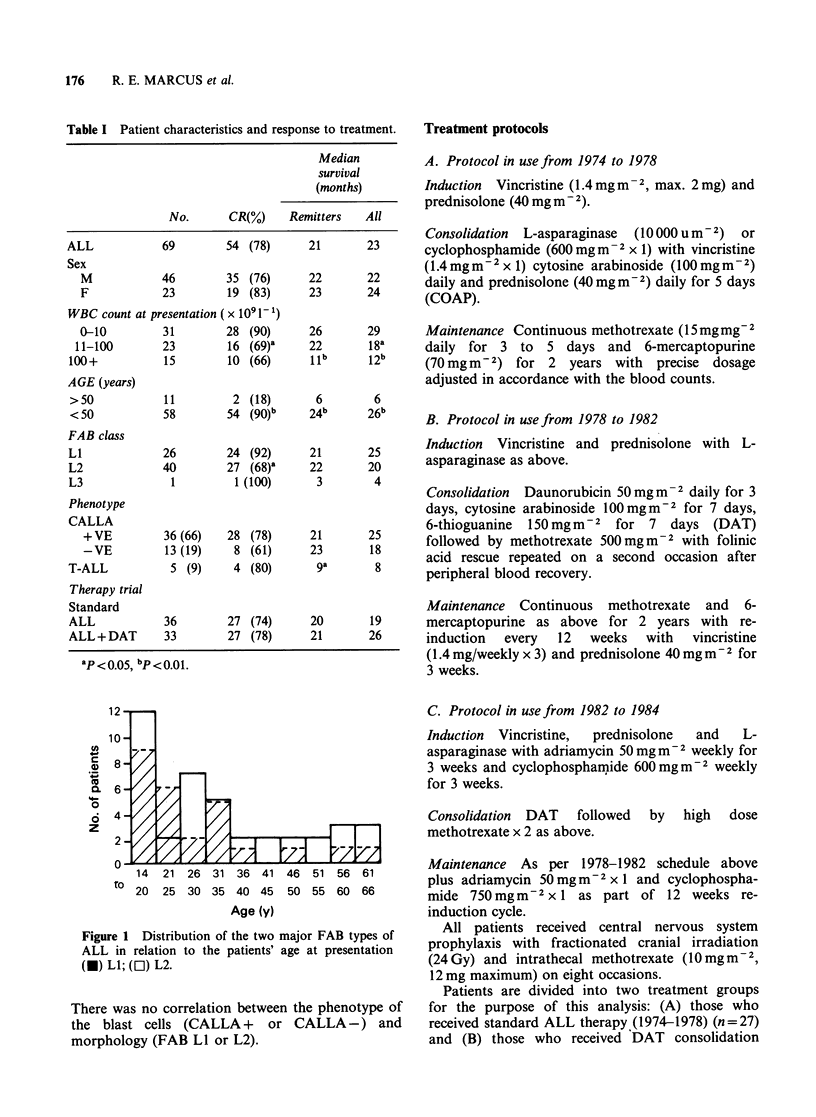

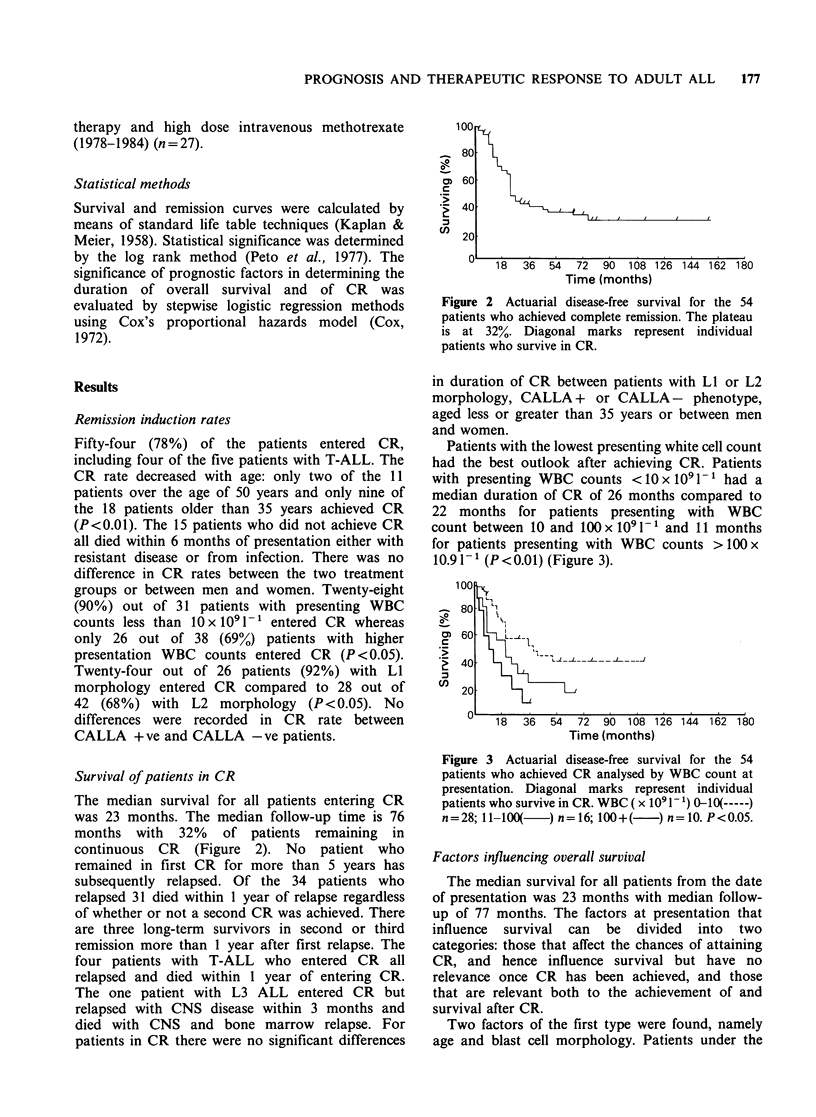

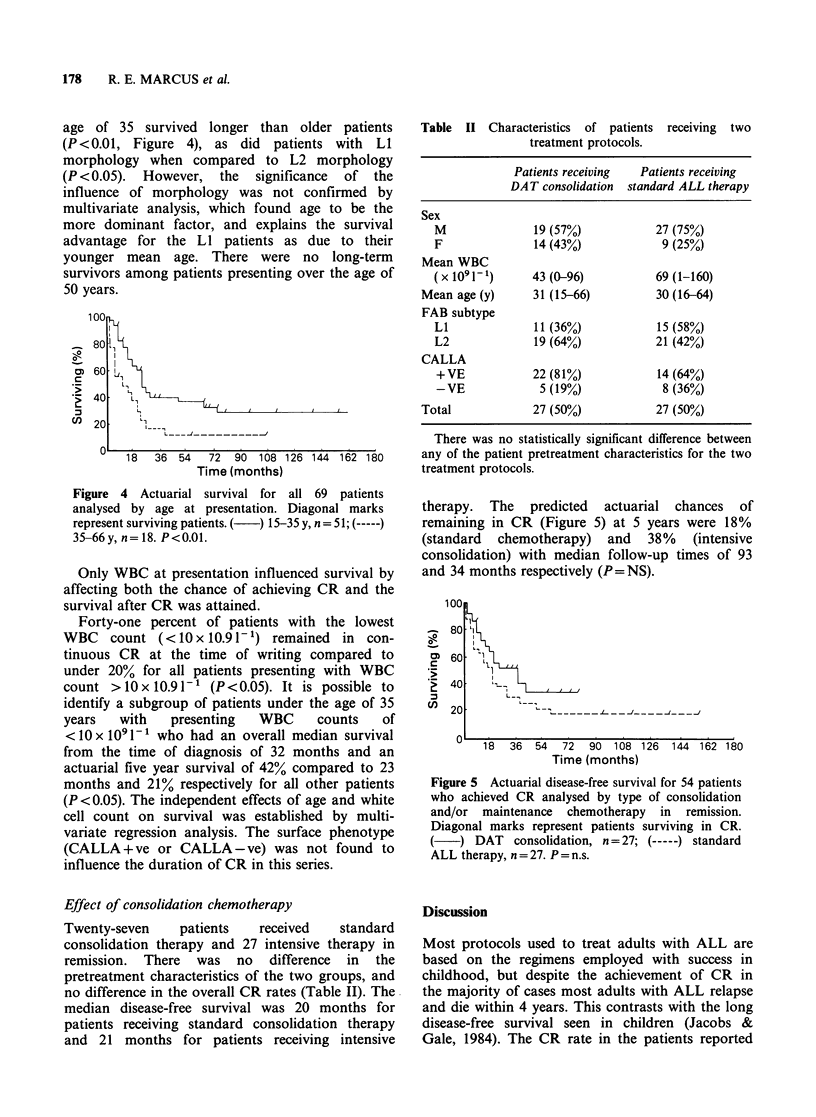

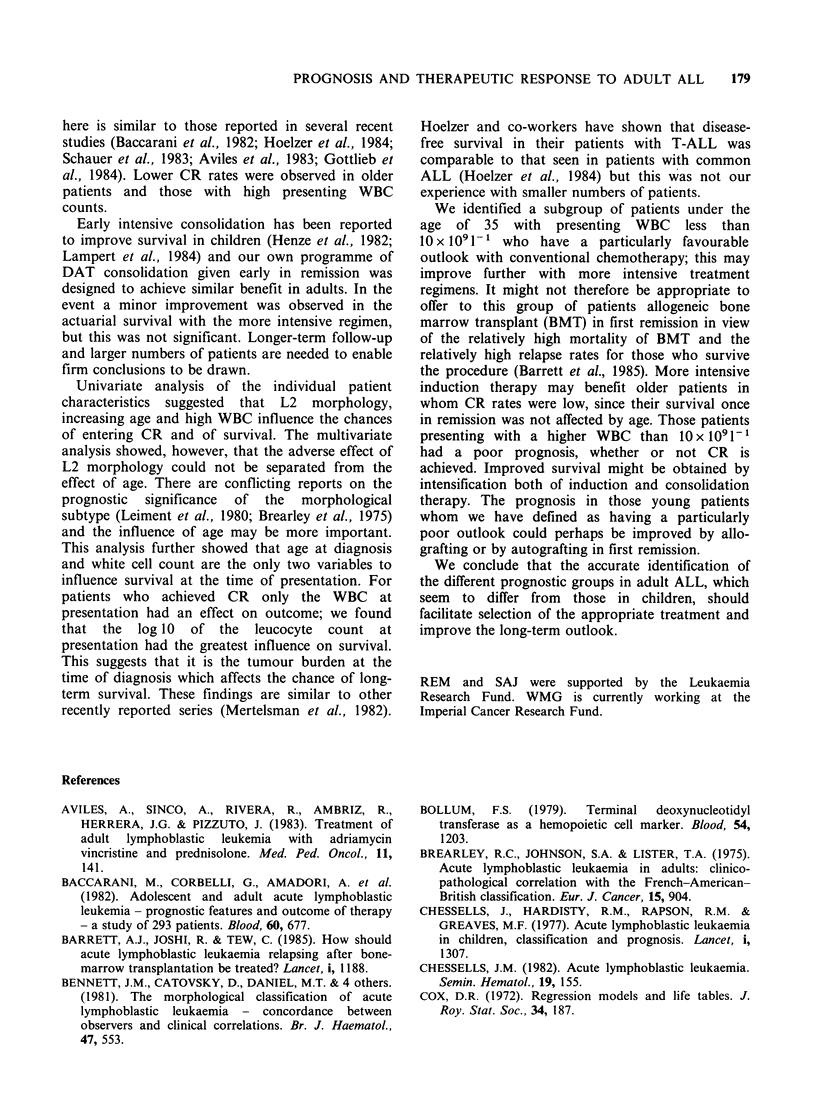

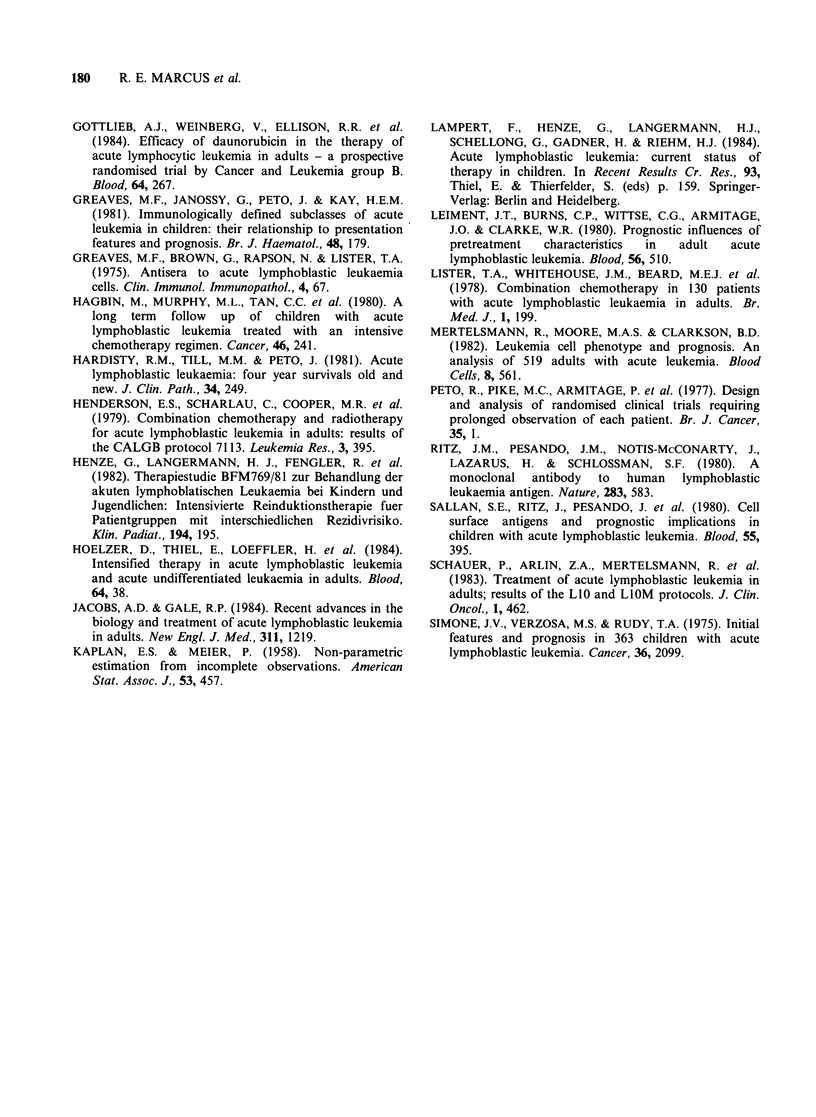


## References

[OCR_00647] Aviles A., Sinco A., Rivera R., Ambriz R., Herrera J. G., Pizzuto J. (1983). Treatment of adult acute lymphoblastic leukemia with adriamycin, vincristine, and prednisone.. Med Pediatr Oncol.

[OCR_00656] Baccarani M., Corbelli G., Amadori S., Drenthe-Schonk A., Willemze R., Meloni G., Cardozo P. L., Haanen C., Mandelli F., Tura S. (1982). Adolescent and adult acute lymphoblastic leukemia: prognostic features and outcome of therapy. A study of 293 patients.. Blood.

[OCR_00662] Barrett A. J., Joshi R., Tew C. (1985). How should acute lymphoblastic leukaemia relapsing after bone-marrow transplantation be treated?. Lancet.

[OCR_00668] Bennett J. M., Catovsky D., Daniel M. T., Flandrin G., Galton D. A., Gralnick H. R., Sultan C. (1981). The morphological classification of acute lymphoblastic leukaemia: concordance among observers and clinical correlations.. Br J Haematol.

[OCR_00674] Bollum F. J. (1979). Terminal deoxynucleotidyl transferase as a hematopoietic cell marker.. Blood.

[OCR_00691] Chessells J. M. (1982). Acute lymphoblastic leukemia.. Semin Hematol.

[OCR_00701] Gottlieb A. J., Weinberg V., Ellison R. R., Henderson E. S., Terebelo H., Rafla S., Cuttner J., Silver R. T., Carey R. W., Levy R. N. (1984). Efficacy of daunorubicin in the therapy of adult acute lymphocytic leukemia: a prospective randomized trial by cancer and leukemia group B.. Blood.

[OCR_00714] Greaves M. F., Brown G., Rapson N. T., Lister T. A. (1975). Antisera to acute lymphoblastic leukemia cells.. Clin Immunol Immunopathol.

[OCR_00708] Greaves M. F., Janossy G., Peto J., Kay H. (1981). Immunologically defined subclasses of acute lymphoblastic leukaemia in children: their relationship to presentation features and prognosis.. Br J Haematol.

[OCR_00719] Haghbin M., Murphy M. L., Tan C. C., Clarkson B. D., Thaler H. T., Passe S., Burchenal J. (1980). A long-term clinical follow-up of children with acute lymphoblastic leukemia treated with intensive chemotherapy regimens.. Cancer.

[OCR_00725] Hardisty R. M., Till M. M., Peto J. (1981). Acute lymphoblastic leukaemia: four-year survivals old and new.. J Clin Pathol.

[OCR_00730] Henderson E. S., Scharlau C., Cooper M. R., Haurani F. I., Silver R. T., Brunner K., Carey R. W., Falkson G., Blom J., Nawabi I. V. (1979). Combination chemotherapy and radiotherapy for acute lymphocytic leukemia in adults: results of CALGB protocol 7113.. Leuk Res.

[OCR_00744] Hoelzer D., Thiel E., Löffler H., Bodenstein H., Plaumann L., Büchner T., Urbanitz D., Koch P., Heimpel H., Engelhardt R. (1984). Intensified therapy in acute lymphoblastic and acute undifferentiated leukemia in adults.. Blood.

[OCR_00750] Jacobs A. D., Gale R. P. (1984). Recent advances in the biology and treatment of acute lymphoblastic leukemia in adults.. N Engl J Med.

[OCR_00761] Lampert F., Henze G., Langermann H. J., Schellong G., Gadner H., Riehm H. J. (1984). Acute lymphoblastic leukemia: current status of therapy in children.. Recent Results Cancer Res.

[OCR_00768] Leimert J. T., Burns C. P., Wiltse C. G., Armitage J. O., Clarke W. R. (1980). Prognostic influence of pretreatment characteristics in adult acute lymphoblastic leukemia.. Blood.

[OCR_00774] Lister T. A., Whitehouse J. M., Beard M. E., Brearley R. L., Wrigley P. F., Oliver R. T., Freeman J. E., Woodruff R. K., Malpas J. S., Paxton A. M. (1978). Combination chemotherapy for acute lymphoblastic leukaemia in adults.. Br Med J.

[OCR_00780] Mertelsmann R., Moore M. A., Clarkson B. (1982). Leukemia cell phenotype and prognosis: an analysis of 519 adults with acute leukemia.. Blood Cells.

[OCR_00786] Peto R., Pike M. C., Armitage P., Breslow N. E., Cox D. R., Howard S. V., Mantel N., McPherson K., Peto J., Smith P. G. (1977). Design and analysis of randomized clinical trials requiring prolonged observation of each patient. II. analysis and examples.. Br J Cancer.

[OCR_00792] Ritz J., Pesando J. M., Notis-McConarty J., Lazarus H., Schlossman S. F. (1980). A monoclonal antibody to human acute lymphoblastic leukaemia antigen.. Nature.

[OCR_00798] Sallan S. E., Ritz J., Pesando J., Gelber R., O'Brien C., Hitchcock S., Coral F., Schlossman S. F. (1980). Cell surface antigens: prognostic implications in childhood acute lymphoblastic leukemia.. Blood.

[OCR_00804] Schauer P., Arlin Z. A., Mertelsmann R., Cirrincione C., Friedman A., Gee T. S., Dowling M., Kempin S., Straus D. J., Koziner B. (1983). Treatment of acute lymphoblastic leukemia in adults: results of the L-10 and L-10M protocols.. J Clin Oncol.

[OCR_00810] Simone J. V., Verzosa M. S., Rudy J. A. (1975). Initial features and prognosis in 363 children with acute lymphocytic leukemia.. Cancer.

